# Supplementary material for: Trends in the prevalence of obesity and estimation of the direct health costs attributable to child and adolescent obesity in Brazil from 2013 to 2022
Source: PLoS One. 2025 Jan 16;20(1):e0308751. doi: 10.1371/journal.pone.0308751 (PMC11737795; doi:10.1371/journal.pone.0308751)
Supplement: S1 Table — (DOCX) [file pone.0308751.s001.docx]

**S1 Table. Data from the primary studies selected for the estimation of the percentage of additional hospitalization costs attributable to childhood obesity (sample size, percentage of additional costs per study, author and year of publication, country of the study, and age-group studied) and the final estimates.**

| **Sample** | **% of additional costs** | **Author, year** | **Country** | **Age-group** |
| --- | --- | --- | --- | --- |
| 518 | 4.68% | Bettenhausen et al, 2015 (47) | United States | Media of 8 years |
| 40,613 | 87.73% | Biener et al, 2020 (4) | United States | 11-17 years |
| 10,804 | 25.61% | Black et al, 2018 (48) | Australia | 6-13 years |
| 496 | 48.70% | Booth et al, 2009 (49) | Australia | 15-19 years |
| 3,508 | 69.15% | Breitfelder et al, 2011 (50) | Germany | Median of 10,2 years |
| 3,528 | 24.61% | Buescher et al, 2008 (15) | United States | 12-18 years |
| 17,942 | 22.55% | Clifford et al, 2015 (51) | Australia | 4-9 years |
| 8,404 | 38.65% | Hampl et al, 2007 (52) | United States | 5-18 years |
| 350 | 63.91% | Hayes et al, 2016 (53) | Australia | 2-5 years |
| 200 | 58.73% | Janicke et al, 2010 (16) | United States | 7-15 years |
| 6,438 | 87.61% | Jerrel et al, 2009 (17) | United States | 0-17 years |
| 4,100,000 | 14.78% | Kompaniyets et al, 2020 (54) | United States | 2-19 years |
| 3,399 | 21.62% | Kuhle et al, 2011 (18) | Canada | 10-11 years |
| 1,643 | 25.05% | Nafiu et al, 2008 (55) | United States | 3-18 years |
| 51,918 | 29.00% | Okubo et al, 2017A (56) | United States | <18 years |
| 38,679 | 2.73% | Okubo et al, 2017B (57) | Japan | 3-8 years |
| 42,698 | 0.95% | Okubo et al, 2018B (58) | Japan | 3-8 years |
| 133,602 | 6.41% | Okubo et al, 2018C (59) | United States | 2-20 years |
| 730 | 19.00% | Ramsey et al, 2020 (60) | United States | 2-18 years |
| 19,613 | 19.90% | Trasande et al, 2009A (19) | United States | 6-19 years |
| 36,583 | 16.55% | Ward et al, 2021 (61) | United States | 6-19 years |
| 14,836 | 1.14% | Wenig et al, 2012 (62) | United States | 2-18 years |
| 1,333 | 11.16% | Wijga et al, 2018 (63) | Netherlands | 14-15 years |
| 774,274 | 22.78% | Woolford et al, 2009 (64) | United States | 2-18 years |
| 5,312,109 |  |  |  |  |
| **Percentage of additional costs** | | | **16.46% (IC 95%: 1.98%-30.94%)** | |
