# Supplementary material for: Trends in the prevalence of obesity and estimation of the direct health costs attributable to child and adolescent obesity in Brazil from 2013 to 2022
Source: PLoS One. 2025 Jan 16;20(1):e0308751. doi: 10.1371/journal.pone.0308751 (PMC11737795; doi:10.1371/journal.pone.0308751)
Supplement: S2 Table — (DOCX) [file pone.0308751.s002.docx]

**S2 Table. Total costs of hospitalizations (Int$) from all causes of children and adolescents by age-group from 2013 to 2022 (National Hospital Information System - SIH/SUS).**

| **Age groups** | **2013** | **2014** | **2015** | **2016** | **2017** | **2018** | **2019** | **2020** | **2021** | **2022** |
| --- | --- | --- | --- | --- | --- | --- | --- | --- | --- | --- |
| **1 to 4 years** | 412,911,855.84 | 412,247,605.01 | 397,738,739.71 | 404,208,566.98 | 417,481,114.14 | 436,874,011.70 | 449,574,245.30 | 314,317,682.03 | 387,279,034.68 | 472,033,763.06 |
| **5 to 9 years** | 247,935,439.23 | 253,113,506.55 | 247,611,822.16 | 241,094,727.71 | 248,609,232.22 | 259,436,243.15 | 269,482,450.46 | 204,210,141.83 | 232,311,078.03 | 272,582,493.95 |
| **10 to 14 years** | 238,956,470.82 | 242,437,002.58 | 234,353,197.95 | 234,421,349.48 | 236,105,785.81 | 239,807,570.41 | 242,349,505.76 | 205,100,700.10 | 224,856,200.72 | 228,616,608.67 |
| **15 to 19 years** | 626,931,085.65 | 645,389,666.44 | 638,211,121.98 | 614,376,577.74 | 614,035,296.70 | 596,731,922.68 | 571,407,538.14 | 512,449,486.03 | 532,381,717.49 | 490,057,227.24 |
| **Total** | 1,526,734,851.54 | 1,553,187,780.58 | 1,517,914,881.80 | 1,494,101,221.91 | 1,516,231,428.87 | 1,532,849,747.94 | 1,532,813,739.66 | 1,236,078,009.99 | 1,376,828,030.92 | 1,463,290,092.92 |
