# Supplementary material for: Trends in the prevalence of obesity and estimation of the direct health costs attributable to child and adolescent obesity in Brazil from 2013 to 2022
Source: PLoS One. 2025 Jan 16;20(1):e0308751. doi: 10.1371/journal.pone.0308751 (PMC11737795; doi:10.1371/journal.pone.0308751)
Supplement: S3 Table — (DOCX) [file pone.0308751.s003.docx]

**S3 Table. Average per capita costs of hospitalizations (Int$) from all causes of children and adolescents by age-group from 2013 to 2022 (National Hospital Information System - SIH/SUS).**

| **Age groups** | **2013** | **2014** | **2015** | **2016** | **2017** | **2018** | **2019** | **2020** | **2021** | **2022** |
| --- | --- | --- | --- | --- | --- | --- | --- | --- | --- | --- |
| **1 to 4 years** | 718.66 | 738.33 | 771.80 | 767.51 | 804.58 | 842.51 | 849.86 | 1,052.65 | 1,044.73 | 962.63 |
| **5 to 9 years** | 659.76 | 690.11 | 719.30 | 705.01 | 730.71 | 750.48 | 754.63 | 868.37 | 919.01 | 812.70 |
| **10 to 14 years** | 749.79 | 780.29 | 789.09 | 813.95 | 831.44 | 844.11 | 849.93 | 964.80 | 1001.13 | 936.43 |
| **15 to 19 years** | 703.35 | 715.38 | 725.52 | 742.75 | 754.07 | 756.36 | 755.18 | 793.84 | 834.64 | 835.48 |
| **Total** | 707.89 | 731.02 | 751.42 | 757.30 | 780.20 | 798.36 | 802.40 | 919.91 | 949.87 | 886.81 |
