# Supplementary material for: Trends in the prevalence of obesity and estimation of the direct health costs attributable to child and adolescent obesity in Brazil from 2013 to 2022
Source: PLoS One. 2025 Jan 16;20(1):e0308751. doi: 10.1371/journal.pone.0308751 (PMC11737795; doi:10.1371/journal.pone.0308751)
Supplement: S4 Table — (DOCX) [file pone.0308751.s004.docx]

**S4 Table. Total number of hospitalizations from all causes of children and adolescents by age-group from 2013 to 2022 (National Hospital Information System - SIH/SUS).**

| **Age groups** | **2013** | **2014** | **2015** | **2016** | **2017** | **2018** | **2019** | **2020** | **2021** | **2022** |
| --- | --- | --- | --- | --- | --- | --- | --- | --- | --- | --- |
| **1 to 4 years** | 574,300 | 557,915 | 515,036 | 526,316 | 518,652 | 518,282 | 528,792 | 298,330 | 370,538 | 490,256 |
| **5 to 9 years** | 375,595 | 366,330 | 343,774 | 341,727 | 339,939 | 345,418 | 356,797 | 234,953 | 252,722 | 335,337 |
| **10 to 14 years** | 317,838 | 309,776 | 296,165 | 287,414 | 283,594 | 283,433 | 284,571 | 212,304 | 224,430 | 243,992 |
| **15 to 19 years** | 885,986 | 897,765 | 876,668 | 824,681 | 812,366 | 786,626 | 754,426 | 643,534 | 635,544 | 584,664 |
| **Total** | 2,153,719 | 2,131,786 | 2,031,643 | 1,980,138 | 1,954,551 | 1,933,759 | 1,924,586 | 1,389,121 | 1,483,234 | 1,654,249 |
