# Supplementary material for: Trends in the prevalence of obesity and estimation of the direct health costs attributable to child and adolescent obesity in Brazil from 2013 to 2022
Source: PLoS One. 2025 Jan 16;20(1):e0308751. doi: 10.1371/journal.pone.0308751 (PMC11737795; doi:10.1371/journal.pone.0308751)
Supplement: S5 Table — (DOCX) [file pone.0308751.s005.docx]

**S5 Table. Original and adjusted prevalence of obesity by age-group from 2013 to 2022 (Sisvan).**

|  |  | **2013** | **2014** | **2015** | **2016** | **2017** | **2018** | **2019** | **2020** | **2021** | **2022** |
| --- | --- | --- | --- | --- | --- | --- | --- | --- | --- | --- | --- |
| **0 to 4 years** | **Original** | 8.56 | 8.91 | 7.56 | 8.11 | 7.13 | 6.91 | 6.95 | 7.39 | 7.58 | 6.35 |
|  | **Adjusted*** | 8.46 | 8.26 | 8.05 | 7.85 | 7.65 | 7.44 | 7.24 | 7.04 | 6.84 | 6.63 |
| **5 to 9 years** | **Original** | 12.38 | 12.53 | 12.98 | 13.55 | 13.33 | 13.23 | 13.19 | 15.82 | 17.84 | 15.81 |
|  | **Adjusted**** | 11.92 | 12.40 | 12.87 | 13.35 | 13.83 | 14.31 | 14.78 | 15.26 | 15.74 | 16.22 |
| **Adolescents** | **Original** | 6.03 | 6.50 | 7.65 | 8.18 | 8.21 | 9.21 | 9.71 | 11.96 | 13.00 | 12.34 |
|  | **Adjusted***** | 5.78 | 6.55 | 7.33 | 8.11 | 8.89 | 9.67 | 10.44 | 11.22 | 12.00 | 12.78 |

* - R^2^ = 0.617

** - R^2^ = 0.686

*** - R^2^ = 0.942
