# Supplementary material for: Trends in the prevalence of obesity and estimation of the direct health costs attributable to child and adolescent obesity in Brazil from 2013 to 2022
Source: PLoS One. 2025 Jan 16;20(1):e0308751. doi: 10.1371/journal.pone.0308751 (PMC11737795; doi:10.1371/journal.pone.0308751)
Supplement: S6 Table — (DOCX) [file pone.0308751.s006.docx]

**S6 Table. Total number of individuals monitored by Sisvan from 2013 to 2022, by age-group.**

|  | **2013** | **2014** | **2015** | **2016** | **2017** | **2018** | **2019** | **2020** | **2021** | **2022** |
| --- | --- | --- | --- | --- | --- | --- | --- | --- | --- | --- |
| **0 to 4 years** | 4,031,834 | 4,181,910 | 4,620,006 | 4,827,198 | 4,777,186 | 5,051,005 | 4,940,387 | 3,778,363 | 4,529,482 | 6,287,802 |
| **5 to 9 years** | 3,027,004 | 3,469,049 | 4,025,308 | 3,779,681 | 3,937,627 | 4,290,916 | 4,494,844 | 2,353,144 | 3,426,261 | 5,227,560 |
| **Adolescents** | 4,281,929 | 4,310,428 | 4,888,991 | 4,697,161 | 4,787,260 | 5,300,728 | 5,126,071 | 3,098,156 | 4,241,412 | 6,428,451 |
| **Total** | 11,340,767 | 11,961,387 | 13,534,305 | 13,304,040 | 13,502,073 | 14,642,649 | 14,561,302 | 9,229,663 | 12,197,155 | 17,943,813 |
