# Supplementary material for: Trends in the prevalence of obesity and estimation of the direct health costs attributable to child and adolescent obesity in Brazil from 2013 to 2022
Source: PLoS One. 2025 Jan 16;20(1):e0308751. doi: 10.1371/journal.pone.0308751 (PMC11737795; doi:10.1371/journal.pone.0308751)
Supplement: S7 Table — (DOCX) [file pone.0308751.s007.docx]

**S7 Table. Proportion of non-hospital, outpatient and medication costs in relation to the hospitalization costs from Ling et al, 2023.**

| Non-hospital costs | Outpatient costs | Medication costs |
| --- | --- | --- |
| 2.86% | 0.80% | 2.35% |
