# Supplementary figures and images for: Trends in the prevalence of obesity and estimation of the direct health costs attributable to child and adolescent obesity in Brazil from 2013 to 2022
Source: PLoS One. 2025 Jan 16;20(1):e0308751. doi: 10.1371/journal.pone.0308751 (PMC11737795; doi:10.1371/journal.pone.0308751)

**S1 Figure. Sensitivity analysis comparing the primary model to different data input scenarios.**


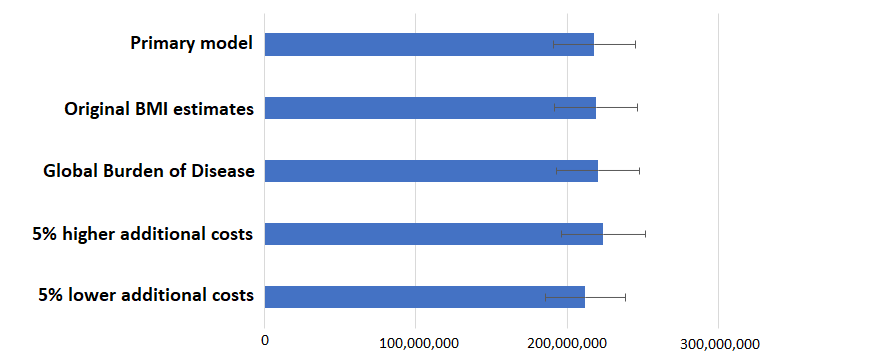

Supplement: S1 Fig — (DOCX) [file pone.0308751.s008.docx]
