# Supplementary material for: Trends in the prevalence of obesity and estimation of the direct health costs attributable to child and adolescent obesity in Brazil from 2013 to 2022
Source: PLoS One. 2025 Jan 16;20(1):e0308751. doi: 10.1371/journal.pone.0308751 (PMC11737795; doi:10.1371/journal.pone.0308751)
Supplement: Attachment — Submitted filename: Point by point responses.docx [file pone.0308751.s009.docx]

Point-by-point responses to the academic editor’s comments and suggestions

**1- The work entitled "Estimation of the direct health costs attributable to child obesity in Brazil from 2013 to 2022" is of great relevance to public health, especially considering the nutritional transition and the obesity epidemic affecting almost everyone, with an alarming impact on children and adolescents. The article is written with clarity and scientific rigor, but after careful reading, I made some suggestions on specific points in the text that require corrections, especially in the methods. After review, the manuscript may be accepted.**

Thank you for your comments and suggestions to improve our manuscript. We have addressed them as follows.

**2- INTRODUCTION – Update references 1, 2, and 3 as the data is quite old (2009 for Brazil and 2016 worldwide) and may not represent the current scenario.**

The references have been updated accordingly.

**3- The authors state that there are no studies on the cost of childhood obesity to the SUS (Brazil’s Unified Health System). Did the authors conduct a literature review to obtain this information?**

We changed the sentence in order to clarify that we have not performed a literature review, but there is a need for studies on the costs of childhood obesity in Brazil.

**4- The study's objective is "...to estimate the direct healthcare costs...," but the introduction does not mention the meaning of "direct healthcare costs." It is important to define this term for a better understanding of the topic.**

**Another issue regarding the objectives: the methods and results present information beyond costs, as the trend of obesity is analyzed. Therefore, the study will have two objectives: the first is to analyze the trend and the second is to estimate direct healthcare costs. I suggest adding this objective to make it consistent with the results. Also, objectives should be included in the abstract.**

Thank you. We adjusted the objectives to incorporate the other results that are presented and included them in the abstract too.

**5- METHODS - The methods need to be clearer and more complete, following the checklist for observational studies (STROBE), which should include the following information: Study design; Setting; Participants; Variables; Data sources/measurement; Bias; Study size; Quantitative variables; Statistical methods. Available at:** [**https://www.strobe-statement.org/checklists/**](https://www.strobe-statement.org/checklists/)

Thank you for the suggestion. As the study is on the economic modeling of the costs to the National Health System, we included the CHEERS Checklist to the supplementary materials instead. The CHEERS (Consolidated Health Economic Evaluation Reporting Standards) 2022 Checklist is more commonly used in cost studies as ours.

**6- I also suggest that the part about trend analysis be more detailed, explaining how this variable is presented by Sisvan, if there is the number of children and adolescents in the system each year. Did the authors consider using the Prais-Winsten regression?**

Thank you. Because the modeling is based on the historical trends found in the children and adolescents monitored by Sisvan, we opted to use the Prais-Winsten regression as suggested. The methodology of the study was also adjusted accordingly.

The number of children and adolescents accompanied by Sisvan was included in the Supplementary Materials (Table S6).

**7- The authors work with Average Annual Percent Change but did not consider the regression p-value to determine if the change was significant. I suggest adding this and including it in the results.**

When we used the Prais-Winsten regression on the Sisvan data, the p-values were included in the results (Table 2).

**8- Another issue that needs justification is the age cutoff of up to 19 years. According to the Brazilian Statute of the Child and Adolescent, a person is considered a child up to twelve incomplete years of age, and an adolescent is between twelve and eighteen years of age.**

Thank you for your comment. Despite the ages in the Statute, we had to consider the adolescents’ age as 10 to 19 years because the data from the Hospital Information System only provides the costs for determined intervals of age (10-14 years and 15-19 years). These age groups are also used in SISVAN for the nutritional status.

**9- DISCUSSION - The authors correctly start the discussion by summarizing the results. However, the trend is not mentioned. If it is in the results, it cannot be ignored. The authors discussed the increasing trend of obesity due to nutritional transition, so it is important to mention this result in the first paragraph and follow the sequence presented in the results: first, the trend, and then the costs.**

Thank you for your comment. We added the trends to the initial part of the discussion.

**10- The authors present data from studies showing adolescents' dietary issues but do not mention the National School Health Survey, which provides this information and is representative of the Brazilian population.**

Thank you one more, the National School Health Survey is very important indeed and has been included in the discussion.

**11- CONCLUSION - Review the conclusion, as it should strictly address the study's objectives. Therefore, the main findings of the study should be added initially, also altering the abstract.**

We reviewed the conclusion and the abstract to strictly address the study’s objectives as recommended.
